# Mapping the Contributing Factors to Missed Nursing Care in Hospital Settings During a Global Health Crisis: A Systematic Scoping Review

**DOI:** 10.1155/jonm/7343469

**Published:** 2025-12-17

**Authors:** Mahsa Pourshaban, Atefeh Allahbakhshian, Majid Purabdollah

**Affiliations:** ^1^ Student Research Committee, Tabriz University of Medical Sciences, Tabriz, Iran, tbzmed.ac.ir; ^2^ Department of Medical-Surgical Nursing, Faculty of Nursing and Midwifery, Tabriz University of Medical Sciences, Tabriz, East Azerbaijan Province, Iran, tbzmed.ac.ir; ^3^ Department of Medical-Surgical Nursing, Faculty of Nursing and Midwifery, Khoy University of Medical Sciences, Khoy, West Azerbaijan Province, Iran

**Keywords:** COVID-19 pandemic, hospital, missed nursing care, nurse, nursing care, scoping review

## Abstract

**Background:**

Little foreknowledge and preparation exist for health‐related crises, and they do not match the magnitude of the problem. During the COVID‐19 pandemic, nursing care in some countries faced more challenges. One of these challenges was missed nursing care. This scoping review aims to identify and map the factors influencing missed nursing care in hospital settings during the COVID‐19 pandemic in studies conducted in developed and developing countries.

**Methods:**

A scoping review was conducted according to methodology recommended by the Joanna Briggs Institute (JBI). We searched five databases—PubMed, Scopus, CINAHL, ProQuest, and Web of Science—as well as the Google Scholar search engine, from December 2019 to July 2025. Keywords of the study were selected according to the Medical Subject Headings (MeSH) and previous research. We included studies in hospital wards that examined missed nursing care and related concepts, specifically those whose data collection periods occurred during the COVID‐19 pandemic. Language restrictions were not applied. The factors were derived inductively, considering conceptual similarities, relevance to the core themes, and similarities in meaning, including aspects related to the missed nursing care model, the developed model derived from it related to the factors considered for missed nursing care, and emerging challenges introduced by COVID‐19. Findings were reported following the PRISMA‐ScR.

**Findings:**

From the 1966 studies, we included 57 articles in the final review. Among them, 50 were cross‐sectional, four were qualitative, two were mixed, and one was quasiexperimental. They were conducted mainly in Iran and the hospital units. Four main themes and nine subthemes emerged (1) work environment (structure, work climate), (2) nurse characteristics (individual and professional, personal), (3) workflow characteristics (intensity, predictability, risk), (4) country (developed, developing). Although the lack of human resources was reported in most studies, it was not the most significant contributing factor.

**Conclusion:**

These findings can inform the development of strategies to address underlying factors affecting workflow, such as nurses’ attitudes and the work environment, thereby enhancing adaptability to future global health crises and serving as a crucial policy foundation for mitigating the missed nursing care during health emergencies.

**Practical Implications:**

These findings not only complement other global research exploring the reasons behind missed cares in nursing but also offer a framework for understanding and anticipating reported instances of missed care, enabling targeted interventions to address them effectively.

**Summary**

• What is already known?
◦ The widespread occurrence of missed nursing care negatively impacts patient care quality.
◦ The COVID‐19 pandemic has underscored the necessity for readiness in health emergencies.
◦ Emerging infectious diseases present challenges that are often underestimated and lack adequate preparation.
◦ Various factors contribute to the phenomenon of missed nursing care.
• What this paper adds?
◦ A broad spectrum of environmental, workflow, individual, and professional factors contributed to missed nursing care during the COVID‐19 pandemic across different countries.
◦ While staffing shortages are commonly noted, they may not be the most significant factor influencing missed nursing care.
◦ The pandemic revealed hidden factors in the work environment, such as attitudes and beliefs, which exacerbated existing issues in healthcare systems crisis.
◦ The level of development of a country influences the magnitude and the nature of the factors that lead to missed nursing care.

## 1. Introduction

In recent years, the phenomenon of missed nursing care (MNC) has garnered significant attention within healthcare systems globally. MNC refers to any essential nursing tasks that are either not performed or postponed [1] and is an important new predictor of patient outcomes [2]. The importance of addressing this issue cannot be overstated, as it directly correlates with patient safety and outcomes, and the efficacy and overall quality of healthcare delivery [3].

Several factors contribute to MNC, including human resource, material resources, and communication. These elements can lead to a compromised ability to provide timely and effective care [1]. Additionally, systemic issues such as poor communication, lack of resources, and inadequate administrative or support staff play critical roles in exacerbating the situation [4]. Understanding these factors is crucial for developing strategies aimed at mitigating MNC, particularly in times of crisis when healthcare systems are under unprecedented pressure [5].

The unprecedented and unfamiliar conditions posed by the novel coronavirus disease significantly impacted the functioning and responses of healthcare and treatment systems [6]. The lack of preparedness for the COVID‐19 outbreak indicates nurses’ tendency to prefer continuing work in the same way as always [7]. As a result, nurses needed to reorganize to respond to a pandemic or a health emergency [8], and the implications of MNC have become even more pronounced [9]. The COVID‐19 pandemic has profoundly impacted nursing care, leading to increased workloads, heightened stress, and a shift in priorities that often results in the neglect of certain nursing duties [10, 11]. Studies have shown that during the pandemic, many nurses reported feeling overwhelmed and unable to meet the demands of their roles, leading to a significant rise in MNC instances [12]. Moreover, during the pandemic, organizational changes, along with feelings of fear and uncertainty, significantly affected nurses and patient care [13]. This crisis has illuminated the vulnerabilities within healthcare systems and highlighted the urgent need for research that explores the dynamics of nursing care in emergency situations. These findings are supported by numerous primary studies conducted during the pandemic [11, 14] that explored frontline nurses’ experiences. In addition, several secondary studies, including systematic and scoping reviews, have synthesized broader evidence on the contributing factors to missed or unfinished nursing care (UNC) [15–17].

The available literature shows that the lack of resources in developing countries makes its allocation to the health sector insufficient. Still, developed countries invest a reasonable budget in the healthcare system [18]. According to the World Health Organization (WHO) report, the health workforce in high‐income countries is 6.5 times larger than that in low‐income countries [118]. In this regard, the amount of MNC is reported higher in low‐income countries compared to high‐income countries, and it varies from country to country [19].

A critical evaluation of five existing literature reveals a range of findings regarding UNC and MNC during the pandemic. The scoping review by Zabaleta et al. [17] included 25 studies conducted up to March 2023, aiming to quantify and categorize the research conducted on UNC [17]. Labrague et al. [20] reviewed only 10 studies conducted until November 2022, employing a systematic review approach to document the types and reasons for MNC [16]. Chiappinotto et al. [21] conducted a systematic review of 5 studies up to May 2021, comparing the occurrence and reasons for UNC during different waves of COVID‐19 [21]. Sist et al. [22] performed an integrative review on UNC of 4 studies conducted until May 2023, summarizing the factors contributing to UNC within the system, unit, nursing management, nurses, and patients [22]. Bayram et al. [15] also conducted a systematic review of 25 studies up to May 2023, primarily focusing on studies from Asian and European countries, reporting emotional status and well‐being of nurses as a primary factor influencing UNC [15].

The findings from various review studies on MNC during the COVID‐19 pandemic indicate that multiple factors influence this phenomenon. While previous research has generally addressed the issues surrounding MNC or UNC, this study delves deeper into the differentiation of these factors across various contexts. It also classifies the contributing factors within hospital settings, thereby enhancing our understanding of the challenges faced by health systems during crises.

Despite offering valuable insights, previous reviews were often limited in scope, with most focusing on specific regions or early pandemic data, which may not fully capture the evolving global context of MNC. These issues may lead to an inadequate reflection of the global status of the phenomenon of MNC during the COVID‐19 pandemic. This highlights the urgent need for a comprehensive scoping review that encompasses a broader range of global evidence and focusing on identifying and mapping the factors influencing MNC during this period in developed and developing countries.

Building upon previous reviews, the present study uniquely aims to map the causal factors influencing MNC in the postpandemic period across both developed and developing countries. By synthesizing global evidence, this scoping review will provide a more comprehensive understanding of how healthcare systems can strengthen nursing capacity, support frontline staff, and enhance preparedness for future health crises.

## 2. Aim

This review aims to identify and map the factors influencing MNC in hospital settings during the COVID‐19 pandemic in studies conducted in developed and developing countries.

## 3. Method

### 3.1. Study Design

**Figure 1:**
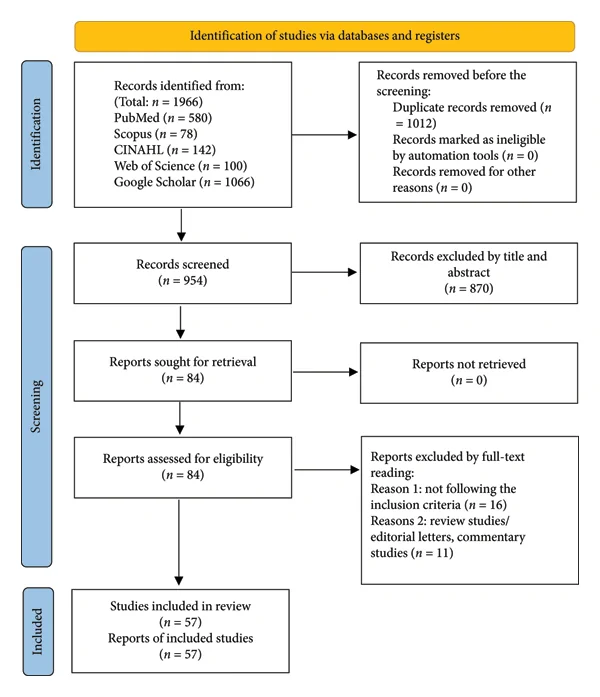
Process of paper selection: diagram flow PRISMA.

A scoping review was conducted according to reporting results and methodology recommended for scoping reviews by the Joanna Briggs Institute (JBI) [23] and reported following to the Preferred Reporting Items for Systematic Reviews and Meta‐Analyses extension for Scoping Reviews (PRISMA‐ScR) [24]. The search results are based on the PRISMA flowchart (Figure 1). Scoping reviews do not primarily focus on conducting analytical comparisons through the aggregation of results from multiple primary sources of evidence. Instead, their aim is to compile and characterize the available evidence and to present a synthesized overview in a transparently visualized manner and to descriptively map and explain the nature of a body of evidence [25]. The decision to use a scoping review was made to encompass all types of evidence, identify research gaps, provide recommendations for further research, assess the breadth of available evidence, and ultimately compile and communicate research findings [23]. The protocol of this scoping review was registered on the Open Science Framework (OSF) to enhance transparency and accessibility (DOI: https://doi.org/10.17605/OSF.IO/X682B, available at https://archive.org/details/osf-registrations-x682b-v1).

### 3.2. Research Question

The Population, Concept, Context (PCC) framework was used to define the research question [26, 27]. Table 1 presents the PCC elements of this study. The guiding question was What factors contribute to MNC in hospital settings during the COVID‐19 pandemic across developed and developing countries?

**Table 1:** Presentation of the PCC strategy applied in the scoping review.

PCC protocol	Review question components
Population	All nurses/nurse assistance
Concept	Research studies that examined missed nursing care during the COVID‐19 pandemic
Context	Quantitative, qualitative, and mixed‐method studies limited to hospital settings in any wards

### 3.3. Search Strategy

PCC criteria were determined to identify relevant articles [23]. Two independent researchers (M.P. and M.PU.) searched various databases, including CINAHL, Web of Science, Scopus, ProQuest and PubMed as well as the Google Scholar search engine, with a time limit from December 2019 to July 2025 to identify articles that may have experienced publication delays, ensuring a comprehensive inclusion of relevant studies. Keywords of the study were selected according to the Medical Subject Headings (MeSH), consultation with the research team, and previous studies. A limited search of MEDLINE was conducted to identify reports on the topic and keywords. To develop a whole search strategy, text words in the title, abstract, and keywords of related reports were checked and used. The search strategy included combining all identified search terms: “omitted nursing care”, “omission care”, “missed care”, “MNC”, “unmet needs”, “undone care”, “unfinished nursing care”, “unfinished care”, “implicitly rationed care”, “implicit care”, “rationing of nursing care”, “rationed care”, “unfulfilled care”, “forgotten nursing care”, “delayed care”, “neglecting care”, “elimination care”, “discontinuous care”, “nursing”, and COVID‐19. Boolean operators “AND” and “OR” and truncation functions appropriate for each database were used. Table 2 presents the detailed search strategy for MEDLINE (via PubMed). All selected reports were searched for additional studies in the reference list. No studies were included as a result of citation searching.

**Table 2:** Search strategy for MEDLINE (via PubMed).

Database 2019–2025	Search terms
PubMed	((“omitted nursing care”[all] OR “omission care”[all] OR “missed care”[all] OR “missed nursing care”[all] OR “unmet needs”[all] OR “undone care”[all] OR “unfinished nursing care”[all] OR “unfinished care”[all] OR “implicitly rationed care”[all] OR “implicit care”[all] OR “rationing of nursing care”[all] OR “rationed care”[all] OR “unfulfilled care”[all] OR “forgotten nursing care”[all] OR “delayed care”[all] OR “neglecting care”[all] OR “elimination care”[all] OR “discontinuous care”[all]) AND (“COVID‐19”[all] OR “COVID 19”[all] OR “2019‐nCoV Infection”[all] OR “SARS‐CoV‐2 Infection”[all] OR “Coronavirus Disease 2019”[all] OR “COVID‐19 Pandemic”[all]) NOT (“systematic review”[all] OR “narrative review”[all] OR “literature review”[all]) AND (”2019/12/01”[PDAT]: “2025/07/01”[PDAT]))

### 3.4. Study Selection

Following the search, the studies were retrieved from online databases and stored using Mendeley software. The studies for this review specified the presence of relevant keywords in the article’s title and abstract, and relevance to the concept of MNC during the COVID‐19 pandemic. We reviewed papers that explored alternative terms for MNC, such as “forgotten nursing care”, “rationing of nursing care”, “MNC”, “care left undone”, and “unfinished nursing care”. The main criteria for considering the articles were predefined inclusion and exclusion criteria. Management of the selection process of the articles includes paying attention to the explanations in all the texts of the articles about the period of data collection related to the COVID‐19 period mentioned in the text of the article and whether the relevant country was affected by the COVID‐19 during the COVID‐19 period or not. In March 2023, the WHO reiterated that COVID‐19 continues to be classified as a global health emergency. However, it was noted that many countries have effectively managed the situation, leading to a gradual reduction in restrictions [28]. The other criteria for eligible studies are shown in Table 3. Categorization of countries was based on international standards, specifically the World Bank classification [29] and the Human Development Index (HDI) published by the United Nations Development Program (UNDP) [30], which classify countries into developed and developing nations based on income levels and socioeconomic development. The selection process is summarized in the PRISMA flow diagram (Figure 1).

**Table 3:** Inclusion and exclusion criteria.

PCC element	Inclusion criteria	Exclusion criteria
Population	All nurses/nurse assistants are included regardless of educational level	Other healthcare workers (doctors, dentists, etc.), nursing students, and patients.
Concept	Contributing factors of Missed/Unfinished/Forgotten/Rationing/omitting, omitted of nursing care during the COVID‐19 pandemic	Studies that did not directly measure contributing factors of missed nursing care and its relation concepts during the COVID‐19 pandemic
Context	Hospital setting in any wards	Nonhospital setting (rehabilitation setting, ambulatory or community/primary care {nursing home})
Article type	Quantitative, qualitative, and mixed‐method studies, gray literature such as doctoral theses	All kinds of reviews, editorial letters, commentary studies, studies of the psychometric assessment of the tool
Publication date/Data collection period	2019–2025/The data collection period was related to COVID‐19 period based on reports from the World Health Organization (WHO), or referenced in the full text or title of articles	Before December 2019/The data collection period was not related to the COVID‐19 period A period in which the determination of COVID‐19 as a pandemic was not in force in that country
Language of publication	All of the languages	—
Text availability	All reported studies have full‐text	Unavailable full text

### 3.5. Data Extraction

Data were extracted using a structured form adapted from the JBI guidelines [23]. After removing duplicates, one researcher (MP) screened titles and abstracts for relevance, and researcher (MPU) double‐checked them. The extraction form was piloted on three studies and refined to align with the research question. Two researchers (MP and AA) independently extracted data, which were then cross‐checked by a third researcher (MPU) to ensure accuracy, consistency, and reproducibility. Disagreements were resolved through discussion. Inter‐rater reliability was assessed using the Kappa coefficient (kappa = 0.75), indicating substantial agreement. Extracted information included study characteristics (authors, publication year, country, period of data collection, unit, aim, study design, sample size), measures, limitations, key findings, and contributing factors of MNC. Tables 4 and 5 summarize these data, with studies grouped by country development level (developed vs. developing), providing a clear rationale for the categorization.

**Table 4:** Summary of studies reported on contributing factors of MNC (MNC) in developing countries during the COVID‐19 pandemic between 2019 and 2025.

Author(s), years, country, data collection period, unit	Aim(s)	Design/sample	Measure(s)	JBI quality, limitation(s) reported in the studies	Key finding(s)	Contributing factors
Safdari et al., 2023 [34]Iran December 2020‐February 2021 COVID‐19 unit	To explain the causes of MNCs during the COVID‐19 pandemic	Qualitative design Purposive sampling Nurses (*n* = 14)	Semistructured interviews	High Low sample size, Impossibility of observing the care process	The increasing demand for care caused by the pandemic and the problems of the work environment have led to the failure of nurses to provide complete, correct care, and sometimes to miss parts of care to patients	1‐Unfulfilled care, 2‐Care at improper times, 3‐Incomplete care, 4‐Incorrect care ‐Lack of resources ‐Communication/teamwork failure ‐Changes in the work environment ‐Adherence to the nursing process ‐High workload ‐Fear of contamination and fatigue of nurses ‐Disproportion between nurses and patients
Safdari et al., 2022 [35] Iran December 2020‐February 2021 COVID‐19 unit	Explaining the factors forming MNCs during the COVID‐19 pandemic	Content analysis Purposive sampling Nurses (*n* = 14)	Depth, semistructured interviews	High Did not tend to express missed care, nongeneralizability, inability to observe the care process and conduct focus groups to generate data	Unpredictable, varying, and stressful work environments, limited human and financial resources, and special characteristics of the disease and infected patients are major challenges for nurses in providing adequate, effective, efficient, and timely care	1‐Care‐related factors 2‐Disease‐related factors 3‐Patient‐related factors 4‐Organization‐related factors
Hosseini et al., 2023 [60] Iran Summer of 2020 COVID‐19 unit	To investigate MNC and the reasons for MNC during the COVID‐19 pandemic	Cross‐sectional design Conventional sampling Nurses caring for COVID‐19 (*n* = 90) Nurses caring for non‐COVID‐19 (*n* = 200)	MISSCARE Survey	Moderate A low number of nurses in Corona wards, lack of cooperation of all staff due to the high workload	Nurses’ position in a new situation and unfamiliarity with the disease and its treatment and care process, had a significant impact on the amount of care provided	‐Inadequate staff (85.2%) ‐Urgent patient situations (74.8%) ‐Unbalanced patient assignments (65.9%) ‐Communication reasons ‐Unpredictable situations ‐Responsibility ‐Type of shifts (rotating and 12‐h) ‐Gender (female) ‐Work experience (more than 10 years)
Mehrabian et al., 2023 [68] Iran January‐ March 2021 Nonemergency wards of 7 medical centers	To investigate MNC and its causes and related factors during the COVID‐19 Pandemic	Cross‐sectional design Simple random sampling Nurses (*n* = 326)	MISSCARE questionnaire ENSS questionnaire	Moderate Self‐reported questionnaires, not recording details of health system characteristics or patient‐related factors	69% of nurses reported missed nursing care occasionally, often, or frequently	‐Inadequate number of staff (70%) ‐Unexpected rise in patient volume and/or acuity in the unit (62%) ‐Inadequate number of assistive and/or clerical personnel (59%) ‐Excessive documentation and duplicate recording (51%) ‐Heavy admission and discharge activity (50%) ‐Human resources (53%) ‐Material resources ‐Communication failure ‐Occupational stress ‐Shift type (rotating) ‐Experience (5–15 years) ‐Job position ‐Overtime work

Khajoei et al., 2023 [64] Iran July 10‐September 10, 2020 Medical‐surgical wards, Intensive Care Unit (ICU)	To determine MNC and related factors in COVID‐19 patients	Descriptive cross‐sectional design Proportionate stratified random sampling Nurses (*n* = 201)	Researcher‐made questionnaire	Moderate The questionnaire, which has not been psychometrically tested	The mean MNC‐related factors were (64.84 ± 10.42). The mean score of MNC in internal surgery wards was higher than in intensive care	‐Physical fatigue of staff (79.1%) ‐Lack of psychological and emotional support (74.1%) ‐Fear of getting sick (74.6%) ‐Lack of psychological and emotional support (74.1%) ‐Lack of financial support (72.1%) ‐Lack of timely visits to patients by specialist physicians (74.1%) ‐Lack of consumables for washing and disinfection (70.6%) ‐Lack of suitable space for spacing between patients (72.6%) ‐Type of ward (ICU) ‐Overload work ‐Employment status ‐Age (50–60)
Muhammadi et al., 2023 [69] Iran 2021 Intensive Care Unit (ICU)	To investigate the rate and causes of MNC for COVID‐19	Cross‐sectional descriptive design Purposive sampling Nurses (*n* = 110)	Missed care questionnaire Causes of MNC questionnaire by Blackman et al. [4]	Moderate Limitation of the study to the ICU department	The level of MNC was middle. The mean MNC‐related factors were (84.85 ± 13.38).	‐Staffing shortages (86.61%) ‐Lack of assistants or secretaries (74.77%) ‐Heavy admission and discharge activity (67.66%) ‐Unexpected rise in patient volume (62.50%) ‐Compulsory overtime (63.39%) ‐Urgent patient situations (60.00%) ‐Human resource (mean = 46.25) ‐Material resource (mean = 19.91) ‐Communication (mean = 18.48)
Farzanehfar et al., 2023 [55] Iran December 2021‐June 2022 Various Hospitals	To determine the relationship between nursing stresses and MNC during the COVID‐19 pandemic	Cross‐sectional design Stratified quota sampling Nurses (*n* = 268)	Forgotten Nursing Care questionnaire ENSS questionnaire	Moderate The large number of questions in the questionnaires	Age, sex, shift in marital status, and position on forgotten nursing care were not statistically significant	‐Nursing stress (60.45%) ‐Employment type ‐Have more children ‐Treatment uncertainty
Amiri Amjad et al. 2023 [46] Iran Jan 2019 ‐ Jul 2020 Medical‐Surgical wards	To investigate the relationship between missed care, work environment, and patient safety culture	Cross‐sectional design Random sampling method Nurses (*n* = 344)	Demographic data form PES‐NWI HSPSC MISSCARE survey (Section A)	Moderate The mental condition of nurses during the COVID‐19 pandemic affects answering the questions	There is no significant relationship between resource adequacy and MNC	‐Patient safety culture ‐Work environment (participation, planning, support, nurse‐physician relations) ‐Age (low) ‐Work experience (low (
Naseri, 2023 [71] Iran 2022 Hospitals	To ascertain, from the perspective of the nurses since the coronavirus pandemic	Cross‐sectional design Random sampling method Nurses (*n* = 300)	Missed care questionnaire Causes of MNC questionnaire by Blackman et al. [4]	Moderate The concern of several nurses about the fear of reprimand and informing the supervisor about the MNCs done by them	The mean score of all the related factors with MNC was 3‐4.	‐Lack of nursing staff (most significant) (mean = 3.84) ‐Emergencies situations (mean = 3.68) ‐Unexpected rise in patient volume (mean = 3.66) ‐A significant number of tasks associated with patient admission and discharge (mean = 3.68)
Pourmovahed et al., 2022 [38] Iran 2021 Neonatal Intensive Care Unit (NICU)	To determine the effect of teamwork training on MNCs during COVID‐19	Quasi‐experimental design Census method Sampling Nurses (*n* = 20) Before/After	MISSCARE Survey	Moderate Due to the lack of willingness of nurses in the study, simulation of different situations and role‐playing by nurses was not conducted	Lack of familiarity with equipment and procedures, knowledge about job descriptions, and the patient’s need for care did not affect missed nursing care.	‐Large volume of activities related to admission and discharge (95%) ‐Teamwork failure ‐Lack of time ‐Lack of nursing staff ‐Large amount of information to be recorded
Dadgari et al., 2023 [33] Iran April‐December 2021 Pediatric ward	To explain the perceptions of pediatric nurses about missed care	Content analysis Purposive sampling Nurses (*n* = 15)	In‐depth semistructured interviews	High Some of the participants might not have mentioned certain issues of missed care due to organizational considerations	Four main categories emerged: missed family‐centered care, unsafe care, predisposing factors, and adverse outcomes	‐Organizational factors (the large number of patients, low number of nurses, lack of time for completion of documents, nonstandard nursing reports, lack of medicines, nonstandard shift delivery, poor teamwork, care without nursing process, and leaving care to mothers) ‐Individual factors (lack of job engagement, not showing sympathy, inexperience, weak work conscience, low care sensitivity, insufficient knowledge, disinterest in nursing, ethnic bias, and dissatisfaction with the employment status)
Maghsoud et al., 2022 [66] Iran October to December 2020 4 hospitals	To investigate the mediating role of implicit rationing of nursing care, job satisfaction, and emotional exhaustion in the relationship between workload and quality of nursing care	Cross‐sectional design Convenience sampling Nurses (*n* = 311)	Demographic information questionnaire NASA‐TLX BERNCA MSQ MBI GNCS	Moderate Generalizability Self‐report questionnaires Recall bias Methodology bias	Three variables of implicit rationing of nursing care, job satisfaction, and emotional exhaustion mediate the relationship between workload and quality of nursing care.	‐Workload ‐Job satisfaction ‐Emotional exhaustion
Fouladi et al., 2024 [57] Iran July 11–October 30, 2021 Internal, surgical, special care, emergency, and pediatric departments	To predict MNC based on moral sensitivity and care‐related factors	Cross‐sectional‐analytical design Stratified random sampling Nurses (*n* = 345)	L‐MSQ Kalish’s missed nursing care Blackman’s missed care‐related factors	Moderate Generalizability	The dimensions of moral sensitivity and related factors of MNC such as human, material, and communication resources are predictors of MNC	‐Moral sensitivity ‐Age ‐Work experience ‐Overtime ‐Second job ‐Education degree ‐Type of unit
Nobahar et al., 2023 [72] Iran June to October 2022 Intensive Care Unit (ICU)	To determine the relationship between teamwork, moral sensitivity, and MNC	Cross‐sectional design Nurses (*n* = 200)	MISSCARE Survey Demographic checklist T‐TPQ L‐MSQ	Moderate Cross‐sectional design Self‐report questionnaire Generalizability	Nurses with a higher understanding of teamwork had higher moral sensitivity and a lower rate of missed care.	‐Teamwork ‐Moral sensitivity ‐Clinical experience ‐Age
Rahmah et al., 2023 [36] Indonesia February–April 2022 Hospitals	To examine the implications of continuous professional development and its impact on MNC in hospitals during the COVID‐19 pandemic	Sequential explanatory mixed design Qualitative sample size: Nurses & head nurses (*n* = 29) Quantitative sample size: Nurses (*n* = 181)	MISSCARE surveys Semistructured interviews & focus group discussions	Moderate Response bias in the data	The rate of all the reasons for missed nursing care was 62–67.5%	‐Inadequate shift‐to‐shift handoff (67.5%) ‐Inadequate number of staff (67.25%) ‐Communication breakdown within the nursing team (66–67.5%) ‐Urgent patient situations (66.75%)
Utomo et al., 2022 [82] Indonesia April 15^th^–July 25^th^ 2021 Neonatal Intensive Care Unit (NICU)	To evaluate the NICU nurse workload, the frequency of missed NICU nursing care, and the other contributing factors associated with missed NICU nursing care	Cross‐sectional design Nurses (*n* = 48)	NASA‐TLX MISSCARE Survey	Moderate A limited size of samples Memory bias Social aspect bias	There was no significant correlation between the total nurse workload and the frequency of any missed nursing care	‐Urgent patient situations (97.92%) ‐Unexpected rise in patient volume and/or acuity in the unit (95.83%) ‐Inadequate number of staff (85.42%) ‐Supplies/equipment not functioning properly (72.72%) ‐ Supplies/equipment not available when needed (64.58%) ‐Tension or communication breakdown with other ancillary/support departments (62.5%)
Khrais et al., 2023 [14] Jordan March‐May 2021 8 hospitals	To examine the effect of perceived organizational support, accountability, and nurses’ characteristics on MNCs under the impact of COVID‐19	Cross‐sectional design Convenience sampling Nurses (*n* = 536)	MISSCARE survey POS scale Accountability Index–Individual Referent	Moderate The sampling method was convenient, generalizability is limited, data were collected online, with a response rate of 63%	‐Communication problems had the highest impact on missed nursing care ‐Accountability and perceived organizational support were the most significant predictors of missed nursing care	‐Communication reasons (mean = 2.8) ‐Labor reasons (mean = 2.7) Material recourses reasons (mean = 2.6) ‐Urgent patient situations (mean = 2.9) ‐Experience ‐Satisfaction with income ‐Perception of accountability ‐Perception of organizational support
Alfuqaha et al., 2023 [44] Jordan November 2019‐ May 2020 Medical surgical ward	To compare the perception of nurses about missed care for the patients before and during the COVID‐19	Comparative cross‐sectional design Nurses (*n* = 260)	MISSCARE survey Job satisfaction items Absence levels The plans of leaving the current position item	Moderate Self‐reported questionnaires, Single setting, Generic instrument	MNC increased during COVID‐19 (22.8%–32.6%). Nurses during the COVID‐19 pandemic were more satisfied, had lower absenteeism, and had lower turnover rates	‐Inadequate number of staff (Mean = 3.68) ‐Labor resources reasons (mean = −3.26) ‐Material recourses reasons (mean = 3.19) ‐Communication reasons (mean = 3.02) ‐Clinical experience (1–5 years) ‐Shift type (B) ‐Age (35–44)
Albsoul et al., 2023 [43] Jordan March and July 2021 10 acute care hospitals	To identify the perceptions of registered nurses for MNC To identify differences in the reported reasons for MNCs across three healthcare sectors	Cross‐sectional design Convenience sampling Nurses (*n* = 672)	MISSCARE Survey tool (Section B)	Moderate Cross‐sectional study design Generalizability Self‐reporting and Response bias Generic instrument	The reasons for MNC (labor, material, communication) were up to 50%. Communication issues were more important to missed nursing care in universities than in public and private hospitals.	‐Patient’s condition worsening (87.2%) ‐Inadequate number of staff (80.1%) ‐Inadequate number of assistive personnel (77.1%) ‐Unexpected rise in patient volume and/or acuity on the unit (73.1%) ‐Unbalanced patient assignments (72%) ‐Interruptions/Multitasking (66.8%) ‐Physical and emotional exhaustion (63.5%) ‐Inadequate supervision of nursing assistants (63.1%) ‐Inadequate support from leadership (60.1%)
Albsoul et al., 2023 [43] Jordan March and July 2021 10 acute care hospitals	To examine the association between the practice environment and MNC	Cross‐sectional design Convenience sampling Nurses (*n* = 672)	MISSCARE Survey tool (Section A) PES‐NWI	Moderate Use of a cross‐sectional study design Self‐reported data only by RNs and the risk of social desirability and recall bias Generalizability	About the PES‐NWI, mean values were above 2.5 for all 5 subscales, indicating that these items were favorable in the involved hospitals.	‐Practice environment elements (personnel, time, support, specialty) ‐Nurses’ participation in hospital affairs (2.59) ‐Quality of care
Albsoul et al., 2023 [43] Jordan March and July 2021 Medical and Surgical wards	To examine the perceptions of Jordanian nurses toward the amount and types of MNC and differences in missed care items between public, private, and university hospitals	Cross‐sectional design Convenience sampling Nurses (*n* = 672)	MISSCARE Survey tool (Section A)	Moderate Use of a cross‐sectional study design Self‐reported data only by RNs and the risk of social desirability and recall bias Generalizability The timing of the data collection	Nurses working in public hospitals were more likely to miss care than those working in private and university hospitals	‐Age ‐Number of patients under care
Mainz et al. 2023 [67] Jordan February‐ April 2022 Critical Care Units (CCU)	To assess the level of job satisfaction and MNC among Jordanian nurses and their association with demographic variables	Cross‐sectional design Convenience sampling Nurses (*n* = 180)	MISSCARE Survey Sociodemographic data form NWSQ	Moderate Low sample size Generalizability Self‐report questionnaire	The level of job satisfaction was moderate MNC was a low‐level A statistically significant weak negative correlation was found between nurses’ job satisfaction and MNC score	‐An unexpected rise in patient volume and acuity on the unit {23.8%} ‐Heavy admission and discharge activity {21%}) ‐Teamwork (the caregiver being off the unit or unavailable {24.3%}) ‐Material resources (unavailability of medications {21.5%})
Nantsupawat et al., 2023 [70] Thailand August‐ October 2022 12 general hospitals	To determine the relationship between nurse burnout, MNC, and care quality following the COVID‐19 pandemic	Cross‐sectional correlational design Nurses (*n* = 394)	MISSCARE survey Emotional Exhaustion (EE) subscale Quality of care reported by nurses	Moderate Cross‐sectional study design Self‐report instruments	The burnout is associated with MNC and poor quality of care following the COVID‐19 pandemic	‐Occupational burnout ‐Anxiety, fatigue, a lack of concentration, and sleeping problems ‐Poor quality of care
Labrague et al., 2022 [20] Philippine 2021 14 hospitals	Identified factors that contributed to MNC and nurse‐assessed quality of care during the COVID‐19	Cross‐sectional design Random cluster sampling Frontline nurses (*n* = 295)	MISSCARE Survey SCS single‐item measure for the overall quality of nursing care	Moderate Generalizability of findings Self‐report measures Response bias	MNC measure was low. Frontline nurses tended to miss clinical aspects of nursing care during the pandemic. In small hospitals, MNC is less reported. A more positive safety culture leads to less MNC	‐Job role (staff nurses) ‐Facility size (large hospital) ‐Nurse staffing levels ‐Patient safety culture ‐PPE adequacy ‐Perception of staff adequacy
Ferreira, 2022 [56] Brazil July–August 2021 Intensive Care Unit (ICU)	To analyze the care omitted from the perspective of the nursing team in a care unit for patients with COVID‐19 and their reasons	Cross‐sectional design Nurses (*n* = 71)	The MISSCARE Survey ‐BRASIL instrument	Moderate The data collection period was not in the increase in cases and admission of patients with COVID‐19 to the investigated hospital Nonspecific instrument in the context of a pandemic	Nursing technicians had higher frequencies in the reasons indicated for omission during care	‐Material resources (lack of equipment {88.9%} and medicines {92.6%}) ‐Unexpected increase in the volume and/or severity of patients (88.9%) ‐Team professionals did not assist when it was needed (85.2%) ‐Urgent patient situations (77.8%) ‐A large number of admissions and discharges (77.8%) ‐Inadequate number of personnel (77.8%) ‐Tension/conflict or communication problems/nursing/medical team (70.4%–74.1%) ‐Lack of standardization for carrying out procedures/care (70.4%) ‐Workplace of employees
Alsalem et al., 2023 [45] Saudi Arabia January‐ March 2023 Neonatal Intensive Care Unit (NICU)	To explain the dimensions and related factors of MNC	Cross‐sectional design Nonprobability convenience sampling Nurses (*n* = 151)	MISSCARE survey	Moderate Cross‐sectional study design Self‐administered questionnaire	The primary reasons for missed care were the shortage of nursing staff and unbalanced patient assignments. Missed nursing care negatively affects job satisfaction and correlates positively with nurses’ intentions to quit.	‐Shortage of nursing staff (significant) ‐Unbalanced patient assignments ‐Inadequate equipment, supplies ‐Breakdowns in communication ‐Satisfaction with the load of work in the unit ‐Intention of leaving current position ‐Days or shifts missed in the past 3 months
Muhammadi et al., 2022 [69] Saudi Arabia June 1, 2021, and September 30, 2021 Inpatient wards	To explore the most common types and reasons behind MNC and its predictors in a unique multinational nursing workforce	Cross‐sectional design Convenience sampling Nurses (*n* = 604)	MISSCARE survey	Moderate Cross‐sectional study design Online questionnaire Generalizability	Missed nursing care is in Saudi Arabia significantly lower than the international rates	‐Inadequate number of staff (significant) (Mean = 3.53) ‐Inadequate number of assistive and/or clerical personnel (Mean = 3.36) ‐Unexpected rise in patient volume and/or acuity on the unit (Mean = 3.29) ‐Heavy admission and discharge activity (Mean = 3.15) ‐Intention to leave ‐Job satisfaction
Mingude et al., 2022 [50] Ethiopia April 2021 medical, pediatric, surgical, and gynecology wards	To assess the magnitude, reason, and associated factors of MNC	Cross‐sectional design Random sampling Nurses (*n* = 315)	MISSCARE survey	Moderate Cross‐sectional design Self‐administered questionnaire Did not include patient feedback	The incidence of MNC was relatively low	‐Unexpected rise in patient volume (88.25%) ‐Inadequate number of nursing staff (87.94%) ‐Labor resource (85.4%) ‐Material resource (84.55%) ‐Educational level (Diploma) ‐Experience (6 m‐2 yrs) ‐Worked with day and night shift ‐Worked over time > 400 h in the past 3 months ‐Dissatisfaction in the nursing profession
Özdelikara and Yaman, 2021 [75] Turkey February‐ April 2021 Hospitals	To determine the amount and causes of health anxiety and MNC needs of nurses working during the pandemic	Cross‐sectional design Snowball sampling Nurses (*n* = 200)	MISSCARE Survey Descriptive Form	Moderate Self‐reports on the digital platform Generalizability	No statistically significant correlation was found between MISSCARE sub‐dimensions and health anxiety scores.	‐Inadequacy of the number of staff (84%) ‐Unexpected increase in the number of patients and/or their density in the working unit (80.5%) ‐Emergency patient situation (74.5%)
Özer and Sari, 2024 [78] Turkey June to December 2021 Pediatric wards	To assess the prevalence of MNC in pediatric clinics, identify its causes, and explore its relationship with nurses’ job satisfaction	Cross‐sectional design Nurses (*n* = 185)	MISSCARE SURVEY ‐ Pediatric Version MSQ	Moderate Generalizability	Job satisfaction among nurses decreased as incidents of missed care increased	‐Insufficient number of auxiliary personnel” (57.3%) ‐Insufficient number of nurses (51.9%) ‐Lack of availability of tools/equipment when needed (31.4%) ‐Lack of cooperation among team members (29.2%) ‐Working patterns (24‐h shifts) ‐Non job satisfaction (62.2%) ‐Gender (male) ‐Type of shift (mixed) ‐Number of patients cared ‐Degrees (master)
Özer and Sari, 2024 [78] Turkey 30 January‐ 30 June 2021 Hospitals	To examine the relationship between MNC and conscientious intelligence	Descriptive correlational design Convenience sampling Nurses (*n* = 514)	MISSCARE Survey‐Turkish Demographic checklist Conscientious intelligence scale	Moderate Cross‐sectional design Self‐report questionnaire (social desirability) Generalizability	Missed nursing care decreased as nurses’ conscientious intelligence increased	‐Conscientious intelligence ‐Gender ‐Education level ‐Satisfaction ‐Patient‐to‐nurse ratios

*Note:* SARS‐CoV‐2: severe acute respiratory syndrome‐COVID‐2; PES‐NWI: Practice Environment Scale of the Nursing Work Index; BERNCA: Basel Extent of Rationing of Nursing Care; NASA‐TLX: NASA‐task load index; T‐TPQ: TeamSTEPPS Team Perception Questionnaire; STEPPS: Team Strategies and Tools to Enhance Performance and Patient Safety.

Abbreviations: ENSS, Expanded Nursing Stress Scale; GNCS, Good Nursing Care Scale; HSPSC, Hospital Survey on Patient Safety Culture; HWEAT, Healthy Work Environment Assessment Tool; L‐MSQ, Lützén Moral Sensitivity Questionnaire; MBI, Maslach Burnout Inventory; MN**C:** missed nursing care; MNCS, Missed Nursing Care Scale; MSQ, Minnesota Satisfaction Questionnaire; NWSQ, nursing‐workplace satisfaction questionnaire; POS, Perceived Organizational Support; PPE, Personal Protective Equipment; SCS, Safety Climate Scale.

**Table 5:** Summary of studies reported on contributing factors of MNC (MNC) in developed countries during the COVID‐19 pandemic between 2019 and 2025.

Author(s), years, country, data collection period, unit	Aim(s)	Design/sample	Measure(s)	JBI quality, limitation(s) reported in the studies	Key finding(s)	Contributing factors
Falk et al., 2022 [54] Sweden Second wave (November 2020) & third wave (May 2021) Critical Care Unit (CCU)	To describe and evaluate reported MNC in the critical care context during different phases of the COVID‐19	Comparative cross‐sectional design Nurses (*n* = 134) Response rate 20.36%	MISSCARE Survey Two specific questions on the perception of patient safety	Moderate Low response rate, low sample sizes, self‐report measures The MISSCARE survey is a generic instrument	Safety and quality of care were good. Medications and supplies/equipment were available. RNs were satisfied with the level of teamwork and perceived the staffing was adequate 50% of the time or more	‐Inadequate number of staff (97.4% & 93.2%) ‐Unexpected rise in patient volume and/or acuity in the unit (92.1% & 89.2%) ‐Urgent patient situations (78.9% & 81.2%) ‐Unbalanced patient assignments (55.3% & 62.2%)
Nymark et al. 2022 [73] Sweden May‐June 2020 Intensive Coronary Care Units (ICU)	To evaluate MNC and patient safety during the first wave of the COVID‐19 pandemic	Cross‐sectional Design Registered nurses (*n* = 43) Nurse assistants (*n* = 59)	MISSCARE Survey Two specific questions on the perception of patient safety	Moderate Low sample sizes Low response rate (18%) Self‐report measures	‐Rated patient safety and quality of care significantly lower during the COVID‐19 pandemic ‐MNCs did not substantially increase during the pandemic	‐Unexpected rise in patient volume and/or acuity in the unit (86.5%) ‐Urgent patient situations (67.6%) ‐Inadequate number of staff (64.1%) ‐Unbalanced patient assignments (56.8%) ‐Heavy admission and discharge activity (55.9%)
Vogelsang et al., 2021 [84] Sweden May–June 2020 Cardiology, heart surgery, vascular surgery, neurology and neurosurgery	To evaluate frequencies, types, and reasons for MNC during the COVID‐19	Comparative cross‐sectional design Registered nurses (*n* = 130) nurse assistants (*n* = 157)	MISSCARE Survey Two specific questions on the perception of patient safety	Moderate Low sample sizes, differences in sample compositions, Limitations of questionnaire items, self‐report measures Collection of samples in nonepidemic peak Low response rate (24.8%)	The level of MNC, quality of care, patient safety, and teamwork satisfaction were similar to before the pandemic with effective management by nursing managers.	‐ Unexpected rise in patient volume (79.8%) ‐ Urgent patient situations (72.9%) ‐ Inadequate number of staff (68.9%) ‐ Unbalanced patient assignments (58.1%) ‐Heavy admission and discharge activity (52.3%)
Amritzer et al., 2024[47] Sweden Between COVID‐19 waves Emergency setting	To describe MNC, quality of care and patient safety assessed by nursing staff	Descriptive cross‐sectional design Nurses and nursing assistants (*N* = 126)	MISSCARE survey–Swedish version Two additional questions about care quality and safety	Moderate The MISSCARE survey was not developed to measure MNC in the emergency setting The self‐reporting format of the instrument Low response rate (22%)	Registered nurses rated most MNC items significantly higher than nursing assistants and viewed both quality and safety and satisfaction with the level of teamwork as worse.	‐Inadequate number of staff *(97.3%)* ‐Unexpected rise in patient volume and/or acuity level (94.7%) ‐Urgent patient situations (90.7%) ‐Unbalanced patient assignments (70.7%) ‐Medications were not available when needed (66.7%) ‐Heavy admission and discharge activity (62.2%) ‐Tension or communication breakdowns with other support departments (58.7%) ‐Inadequate number of assistive personnel (57.5%) ‐Nursing staff did not communicate that care was not done (54.7%)
Cengia et al., 2022 [52] Italy November 2020‐January 2021 Medical and surgical units	To compare the occurrence and the reasons for unfinished care in COVID‐19 and non‐COVID‐19 patients	Comparative cross‐sectional design Nurses (*n* = 479)	UNC Survey	Moderate The effect of stress on the quality of answers Time limit to complete the survey Unbalanced number of participants (90 vs. 200) scale validity	Did not show differences in the occurrence of unfinished care. Reasons for incomplete care showed significantly higher among nurses caring for COVID‐19	‐Priority setting (mean = 2.78) ‐Nurses’ aide supervision (mean 2.58) ‐Communication failure (mean = 2.40)
Chiappinotto and Palese, 2022 [32] Italy May and August 2021 6 hospitals	To investigate reasons for UNC across the whole levels of the nursing service as perceived	Descriptive qualitative approach (content analysis) Purposeful sampling Clinical, manager, and executive nurses (*n* = 29)	Interview	High This study was limited to one large health care, context, pandemic, nursing service	Clinical nurses perceive the reasons for unfinished nursing care in several different elements similar to the perception of ward managers, whereas executive nurses have a different perspective of the reasons.	‐System‐level (Poor support) ‐Unit Level (Inappropriate care environment insufficient material and human resources, ineffective inter‐ and intraprofessional cooperation, ineffective work process, Ineffective shift design, Ineffective models of nursing care delivery) ‐Nurse managers (inadequate nurse manager’s leadership) ‐Clinical nurses (ineffective nurses’ performances, weaknesses in education, poor humanistic view of patient care, ineffective priority‐setting skills) ‐Patients’ levels (increased demand for care, lack of career support).
Bruyneel et al. 2024 [51] Belgium December 2021‐ February 2022 75 Intensive Care Units (ICU)	1. To measure the prevalence of UNC in intensive care units during the COVID‐19 pandemic 2. To examine whether UNC has a mediating role in the relationship between nurse working environment and nurse‐perceived quality of care and risk of burnout	Cross‐sectional design Nurses (*n* = 2183)	UNC Survey PES‐NWI The perception of quality and safety of care Maslach Burnout Inventory scale	Moderate Cross‐sectional study design and lack of causal interpretations self‐reported bias	unfinished nursing care had a mediating effect between adequacy of staffing and resources and perceived quality and safety of care and the burnout risk among ICU nurses	‐The staffing and resource inadequacy(significant) ‐Nurse perceived of quality and safety of care ‐Occupational burnout
Sugg et al., 2021 [37] England 3rd to 26th August 2020 General hospitals Non ventilate patients	To identify views and experiences of nursing staff on necessary nursing care for inpatients with SARS‐CoV‐2 that is omitted or delayed and any barriers to this care	Mixed methods explanatory design Convenience sampling Nurses (*n* = 1062)	A bespoke online series of survey questions	Moderate Absence of a predefined sample size Participant fatigue The samples were only RN nurses	Nurses caring for patients with SARS‐CoV‐2 faced challenges in supporting patients in all three care areas	‐High patient‐to‐nurse ratios ‐Lack of nurse time ‐Severity of patients’ conditions ‐Practice environment ‐Significant workload, fatigue, anxiety, and mental health symptoms among healthcare ‐Wearing PPE ‐Reluctance to spend time with patients for fear of catching SARS‐CoV‐2 ‐Lack of presence from specialized services e.g., physiotherapists ‐Lack of knowledge about SARS‐CoV‐2 ‐Insufficient stock
Zhang et al., 2024 [85] China November to December 2022 Hospital	To analyze the status and relationship between job satisfaction and MNC among frontline nurses in a hospital dedicated to COVID‐19	Cross‐sectional design Convenience and snowball sampling Frontline nurses (*n* = 306)	Demographic profiles questionnaire Job satisfaction scale MISSCARE Survey questionnaire	Moderate Self‐report bias Generalizability Memory deviations of frontline nurses	The most reported reasons for the MNC were urgent patient situations. The rate of each reason for missed care ranged from 18.09% to 55.26%. The job satisfaction was high while their MNC was low. Nurses were working for 4–6 hours per shift.	‐Urgent patient situations (55.26%) ‐Unexpected rise in patient volume and/or acuity on the unit (53.95%) ‐Job satisfaction ‐Type of ward ‐Type of hospital ‐Age (younger) ‐Gender (male) ‐Lacked support from family and friends ‐Working hours per shift
Edmonds et al., 2023 [53] United States January 14‐ February 26, 2021 Labor and delivery units.	To examine the relationships between three missed critical nursing care processes in labor and delivery units with reduced nursing time at the bedside and adequacy of unit staffing during the COVID‐19 pandemic	Cross‐sectional design Convenience sampling Nurses (*n* = 836)	Perinatal Missed Care Survey A researcher‐developed question	Moderate Disable causal inferences Self‐reported data Convenience sample Recall bias Potential social desirability bias Not psychometrically evaluated a researcher‐developed question	One‐third of respondents (*n* = 278, 33.3%) reported that they or nursing staff on their units missed at least one of the three critical nursing care processes.	‐Less nursing time at the bedside ‐Inadequate number of staff (*50%)*
Vincelette et al., 2023 [83] Canada September 2021 Intensive Care Unit (ICU)	To describe the characteristics of omitted nursing care To examine the associations between work environment features, omitted nursing care and nurse‐reported outcomes	Cross‐sectional correlational design Nurses (*n* = 493)	HWEAT ICU‐ONC Perceptions of nurse‐reported outcomes	Moderate Self‐report bias Cross‐sectional design (does not allow for causal inferences)	On average, nurses felt that their work environment was acceptable and that the quality and safety of patient care was good. Omitted nursing care was rare in the ICU for most activities	‐Work environment ‐Negative perceptions about the quality and safety of care
Mainz et al., 2024 [67] Denmark November and December 2020 34 surgical, medical wards	To investigate the prevalence and reasons for MNC and whether nurses’ work experience was associated with MNC	Cross‐sectional design Nurses (*n* = 1241)	MISSCARE survey	Moderate MISSCARE surveys are based on the staff’s perceptions, not observations of the actual care provided	The prevalence of missed nursing care elements rand from 10% to 76%.	‐Inadequate number of nurses *(86%)* ‐Heavy admission and discharge activity (71%) ‐Unexpected rise in patient volume (85%) ‐Urgent patient situations (64%) ‐Work experience (less than 5 years)
Cartaxo et al., 2024 [31] Austria August 2020‐ February 2022 General units	To explore nontrivial configurations of reasons for missed care that are associated with missed care interventions from the perspective of nurses	Cross‐sectional design Qualitative comparative analysis The semiprobabilistic approach based on snowball sampling Nurses (*n* = 401)	MISSCARE‐Austria questionnaire	Moderate Methodological bias (noncausal explanations)	Missed care and the various contextual factors and reasons contributing to its occurrence are embedded in complex adaptive systems and need to be considered through the lens of complexity theory in nursing science	‐High demand for patient care (mean = 4.6) ‐Poor labor resources (mean = 4.6) ‐Difficulties in relationships and communication (mean = 3.0) ‐Poor material resources (mean = 2.7) ‐Nurses’ experience, role, or qualification ‐Inadequate nursing staffing (71.6%)
Sarpong et al., 2023 [77] Australia July‐December 2021 Medical and surgical wards	To determine nurses’ perceptions of the types of MNCs, and reasons and to identify factors predicting	Cross‐sectional descriptive design Nurses (*n* = 204)	MISSCARE survey	Moderate Self‐reported and retrospective survey Precision and generalizability	Labor resources ranked highest for reasons for missed care	‐Labor resources (mean = 3.38) ‐Inadequate number of staff (mean = 3.74) ‐Material resources (mean = 2.70) ‐Communication (mean = 2.35) ‐Heavy admissions and discharges (mean = 3.41) ‐Unexpected rise in patient volume or acuity (mean = 3.51) ‐Urgent patient situation (mean = 3.31) ‐Job satisfaction ‐Role satisfaction ‐Teamwork ‐Working overtime
Kim and Chae, 2022 [65] South Korea May 21 to June 1, 2021 Neonatal Intensive Care Units (NICU)	To examine missed care and its influencing factors	Cross‐sectional design Snowballing method of convenience sampling Nurses (*n* = 118)	MISSCARE survey	Moderate Specifically of instrument Snowballing method	Missed care was affected by nurses’ workload related to the number of patients taking medication	‐Emergency within the unit or deterioration of one of the assigned patients (100%) ‐Various interruptions during work (100%) ‐Increase in the number of patients (98.3%) ‐Shortage of nurses (99.1%) ‐Too many admissions or discharges (99.1%)
Antoszewska and Gutysz Wojnicka. 2024 [48] Poland January and May 2023 Intensive Care Unit (ICU)	To identify the extent to which nursing care is rationed in intensive care units, as well as assess the quality of nursing care, and the level of job satisfaction and its correlation with an assessment of the climate of work safety, teamwork, and a healthy work environment	Cross‐sectional correlational design Nonrandomized sampling Nurses (*n* = 226)	PRINCA Patient care quality and job satisfaction HWEAT Teamwork and Safety Climate (BePoZa)	Moderate Nonrandom selection Attrition	The scores from the questionnaires were negatively correlated with the nursing care rationing scores	‐Work Environment ‐Teamwork and safety climate
Jędrzejczyk et al., 2023 [63] Poland January to October 2022 Internal Medicine wards in 8 hospitals	To examine the factors influencing the rationing of nursing care, nurses’ assessment of the quality of patient care, and their job satisfaction	Cross‐sectional descriptive design Nurses (*n* = 1164)	Demographic data sheet PIRNCA Quality of patient care and job satisfaction questionnaire	Moderate Generalizability	Respondents rarely ration nursing care The quality of patient care and job satisfaction was at a moderate level	‐Work experience of 16–20 years ‐Bachelor’s degree ‐Gender (female)
Tomaszewska et al., 2024 [80] Poland December 2022 and January 2023 Intensive Care Unit (ICU)	To determine the relationship between occupational burnout, job satisfaction, and rationing of care among anesthesia nurses employed in intensive care units	Cross‐sectional design Nurses (*n* = 477)	BERNCA‐R questionnaire Job Satisfaction Scale questionnaire Maslach Burnout Inventory questionnaire	Moderate Generalizability	All the scores obtained exceeded the threshold of 50% of total points, which indicates the presence of occupational burnout at a significant level. The job satisfaction of the nurses was high	‐Occupational burnout ‐Lack of time
Tomaszewska et al., 2021 [79] Poland September and December 2020 Surgical and nonsurgical wards	To assess the rationing of the level of nursing care among nurses	Cross‐sectional design Nurses (*n* = 295)	BERNCA	Moderate Limitations in contact with a wider group of respondents	The higher frequency of rationing nursing care was characteristic of those working on surgical wards	‐Seniority and place of work ‐Type of unit
Jarosz et al., 2022 [61] Poland March–May 2022 Urology department	To assess the level of rationing care, fatigue, job satisfaction and occupational burnout and to assess the relationship between them and age, length of service and the number of jobs	Cross‐sectional design Nurses (*n* = 130)	PIRNCA LBQ SSP SWLS MFIS	Moderate Limit to urological nurses Small sample Online questionnaire Limitations in reaching urological wards related to the SARS‐CoV‐2 pandemic	Care in urology departments is rarely rationed	‐Occupational burnout ‐Job satisfaction ‐Age ‐Work experience ‐Fatigue
Jarosz and Mlynarska, 2023 [62] Poland March–May 2021 Urology departments	To assess the impact of sociodemographic factors (place of residence, forms of postgraduate education, work system, number of patients per one nurse on duty, satisfaction with the financial situation, number of diseases the nurse suffers from) on the rationing of nursing care	Cross‐sectional design Nurses (*n* = 130)	PIRNCA	Moderate Limit to urological nurses Small sample Online questionnaire Limitations in reaching urological wards related to the SARS‐CoV‐2 pandemic	Care in urology departments is rarely rationed	‐Sociodemographic characteristics ‐Job satisfaction ‐Number of diseases the nurse ‐Satisfaction with the financial situation ‐Place of residence
Uchmanowicz et al., 2021 [81] Poland January and May 2020 NICU	To assess the relationship between the rationing of nursing care and professional burnout in nursing staff	Cross‐sectional, observational design Nurses (*n* = 219)	BERNCA‐R MBI	Moderate Generalizability	The level of rationing of nursing care in cardiovascular facilities increases along with emotional exhaustion, depersonalization and job dissatisfaction, and multijobs activity	‐Emotional exhaustion ‐Depersonalization ‐Job dissatisfaction ‐Multijobs activity ‐Age (41–50 years) ‐Job experience (low) ‐Bachelor’s degree
Obregón‐Gutiérrez et al., 2022 [74] Spain May 2020 Acute care (COVID‐19 unit)	To analyze the quality of care provided during the COVID‐19 pandemic, identifying what care has been prioritized and factors that have influenced such care	Cross‐sectional design Convenience sampling Nurses (*n* = 225) (Response rate 91.8%)	Care Left Undone Scale Self‐perceived assessment List identifying feelings List of personal and professional strategies	Moderate N/A	Nurses were able to maintain and increase care quality during the COVID‐19 pandemic by developing personal and professional strategies	‐Physical barriers in the work environment (52.6%) ‐Little time for care (48%) ‐High workload (47%) ‐Lack of knowledge in using protective equipment (48%) ‐Isolation of patients from family (47%) ‐Fear of infecting family members ‐Change of structure ‐Change of care activities ‐Age ‐Experience ‐Specialty ‐perceived independence ‐Personal and professional strategies
Gurkova et al., 2022 [59] Czech Republic Between April and September 2020 Medical and surgical units	To investigate which domains of the work environment were significant predictors of MNC activities during the COVID‐19	Cross‐sectional design Nonprobability sampling Nurses (*n* = 371)	MISSCARE Survey (Part A) PES‐NWI	Moderate Generalizability of findings, self‐report measures, response bias, nonprobability sampling. The cross‐sectional design	The nursing work environment was favorable (63%). The prevalence of MNCs was not higher before the COVID‐19 pandemic (36.3%)	‐Nurses’ perception of nursing foundations for quality of care ‐Satisfaction with their current position ‐Overtime work ‐Perception of favorable work environments ‐Hospital facility level
Gurkova et al., 2021 [58] Czech Republic Between April and September 2020 Medical and surgical units	To examine the differences in reasons for UNC according to the type of hospitals and wards To investigate the relationship between the reasons for UNC and nurses’ job satisfaction	Cross‐sectional design Nonprobability sampling Acute care nurses (*n* = 371)	MISSCARE Survey questionnaire (Part B)	Moderate The generalizability of findings does not reflect the situation in Czech hospitals during the second wave of the pandemic	Human resources and staffing were rated as the most important reasons for unfinished nursing care	‐Human resources (insufficient staffing) ‐Unexpected increase in the number ‐Severity of patients’ conditions ‐Emergencies in patients ‐Hospital type ‐The nurses’ satisfaction with their current position ‐Satisfaction of role as a nurse ‐Satisfaction teamwork
Bartoníčková et al., 2022 [49] Czech Republic January‐ March 2021 Pediatric care units	To examine the association between MNC and the work environment	Descriptive cross‐sectional design Purposeful sampling Nurses (*n* = 138)	MISSCARE Survey‐Ped‐CZ Sociodemographic questions PES‐NWI	Moderate Absence of an evaluation of the CVI index in the validation of the instrument The low number of participating care units Testing the reliability of MISSCARE‐Survey‐Ped‐CZ only in terms of internal consistency	Correlations between missed nursing care and work environment have not been confirmed. The relationship between the work environment and perceived staff adequacy was proved.	‐Frequent interruptions (81.2%) ‐Unexpected rise in patient volume and/or acuity on the unit (73.2%) ‐Urgent patient situation” (66.7%) ‐Unbalanced patient assignments (63%) ‐Tension or communication breakdowns within the medical staff (56.6%) ‐Inadequate number of nurses (53.6%) ‐Tension or communication breakdowns with other services or departments (52.9%) ‐Supplies/equipment not properly functioning when needed (51.4%) ‐Inadequate number of personnel (50.7%)

*Note:* SARS‐CoV‐2: severe acute respiratory syndrome‐COVID‐2, PES‐NWI: Practice Environment Scale of the Nursing Work Index, ICU‐ONC: Intensive Care Unit Omitted Nursing Care instrument, BERNCA: Basel Extent of Rationing of Nursing Care, PRINCA: Perceived Implicit Rationing of Nursing Care; SSP: Work Satisfaction Scale; SWLS: Life Satisfaction Scale.

Abbreviations: HWEAT, Healthy Work Environment Assessment Tool; LBQ, Link Burnout Questionnaire; MBI, Maslach Burnout Inventor; MFIS, Modified Fatigue Impact Scaley; MNC, missed nursing care; MNCS, Missed Nursing Care Scale; PPE, Personal Protective Equipment; UNC, Unfinished Nursing Care.

### 3.6. Quality Appraisal

In this scoping review, the primary goal was to map and describe the existing evidence transparently, following the guidelines of the JBI [23, 25]. In scoping reviews, conducting a critical appraisal of the sources of evidence is frequently bypassed, as the primary aim of such reviews is to systematically describe and visualize a body of literature based on the characteristics and factors specified by the review’s objectives, research questions, and inclusion criteria [25]. Therefore, it is important to emphasize that the quality assessment was not utilized as a criterion for exclusion but rather aimed to assess the overall rigor of the studies included. This approach ensures a transparent evaluation of the existing body of knowledge. Quality appraisal of included studies was conducted using the JBI critical appraisal tools according to the study design. The score was given as follows: each item of the JBI Checklist was answered as Yes, No, Not Applicable, or Not Reported. Studies meeting a predefined threshold across the items were classified as high quality, those meeting fewer items as moderate quality, and those with substantial gaps as low quality. Inter‐rater reliability was ensured by independent assessment by two researchers (M.PU. and A.A.), with disagreements resolved through discussion or adjudication by a third researcher (M.P.). The results of this review are presented in Tables 4 and 5. Quality scores did not affect study inclusion.

### 3.7. Data Analysis

Thematic analysis was used to synthesize data on factors contributing to MNC. Factors contributing to MNC were extracted from the full text, including tables and narrative descriptions. Thematic coding was performed inductively using NVivo. The process included (1) *Open Coding:* Meaningful segments related to potential contributing factors were highlighted and labeled. (2) *Grouping Codes:* Codes were compared across studies and grouped based on conceptual similarity and relevance to the MNC model [1] as well as the expanded conceptual model proposed by Cartaxo et al. [31], which builds upon Kalisch’s framework and incorporates more complex configurations of contributing factors, particularly relevant to the pandemic context [31], and emerging challenges introduced by COVID‐19. (3) *Consensus on Categories:* Three researchers (MP, AA, and MPU) independently developed categories, discussed discrepancies, and finalized them through iterative rounds.

A factor was included if at least two independent studies reported it. This approach ensured that the identified factors were supported by multiple sources. The final causal factors were summarized in tables (Tables 4 and 5), grouped according to the country development level (developed vs. developing), providing a clear rationale for how studies were categorized. The final categories provide a structured overview of causal factors, reflecting their diversity and complexity across different settings. This synthesis allows a clear mapping of factors according to country development level while maintaining the descriptive, nonanalytical focus appropriate for a scoping review.

### 3.8. Ethical Considerations and Dissemination

This article received ethical approval under the code IR.TBZMED.VCR.REC.1404.012. No primary data were collected from research participants for this review. All data were compiled from published peer‐reviewed studies that are already publicly available. However, the following were considered: careful selection of eligible studies, reporting of valid studies, conducting an impartial assessment of eligible studies, integrity and consistency in extracting results and avoiding any bias. The study protocol was reviewed and finalized by the university’s Research Committee before implementation. No substantive changes have been made relative to the approved version, and any future modifications will be documented through the formal registration process.

## 4. Results

### 4.1. Search Results and Data Evaluation

In this scoping review, 1966 studies were retrieved from online databases and stored using Mendeley software. Two of the researchers reviewed the titles and abstracts of the selected articles. After removing duplicate and unrelated titles (*n* = 1882), two researchers independently reviewed the full texts of 84 articles. Twenty‐seven studies were excluded for different reasons [not following the inclusion criteria (*n* = 16), review studies/editorial letters, and commentary studies (*n* = 11)]. Only 57 articles were ultimately eligible for further analysis.

### 4.2. Characteristics of the Selected Studies

Tables 4 and 5 summarize the characteristics of the articles included in this study. Out of the total 57 studies reviewed between December 2019 to March 2025, four were qualitative studies [32–35], two were mixed studies [36, 37], one was quasiexperimental [38], and 50 were quantitative with cross‐sectional design [14, 20, 25, 31, 39–85].

The studies were conducted by authors from 31 developing countries (14 studied Iran (45.16%), 6 studied Jordan (19.35%), 3 studied Turkey (9.67%), 2 studied Indonesia (6.45%), 2 studied Saudi Arabia (6.45%), 1 studied Ethiopia (3.22%), 1 studied Thailand (3.22%), 1 studied Brazil (3.22%), and 1 studied the Philippine (%3.22%)) and 26 developed countries (4 studied Sweden (15.38%), 7 studied Poland (26.92%), 3 studied the Czech Republic (11.53%), 2 studied Italy (7.69%),1 studied Spain (3.84%), 1 study England (3.84%), 1 study China (3.84%), 1 study Belgium (3.84%), 1 study United States (3.84%), 1 study Canada (3.84%), 1 study Denmark (%3.84%), 1 study Australia (3.84%), 1 study South Korea (3.84%), and 1 study Austria (3.84%)).

The context of the studies included the intensive care unit (ICU), pediatric ward, neonatal ICU (NICU), medical, surgical, emergency, COVID‐19 unit, nonemergency wards, critical care units (CCUs), cardiology, urology, gynecology, labor and delivery, cardiology, heart surgery, vascular surgery, neurology, and neurosurgery units. Thirteen studies did not specify the unit or ward where the study was conducted (only mentioned the hospitals or their type) (Tables 3 and 4). The total number of frontline and assistant nurses studied in these articles ranged from 14 to 2183. The most reported limitations in the conducted studies include methodological bias (noncausal study design), the measurement instruments used and their nonspecificity for the COVID‐19 era, the self‐reporting nature of the tools (social desirability and recall bias), and generalizability.

### 4.3. Most Frequently Reported Contributing Factors of MNC During the COVID‐19 Pandemic

**Figure 2:**
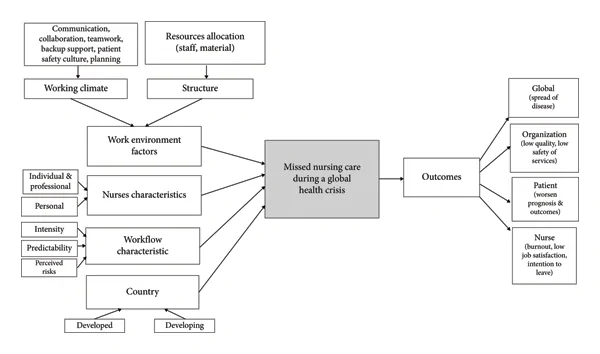
The underlying conceptual model used to inform the study.

The contributing factors of MNC during the COVID‐19 pandemic were thematically categorized in four main themes and nine subthemes. A conceptual model of contributing factors of MNC is shown in Figure 2. The most frequent contributing factors reported in the eligible studies, if reported as percentages or mean, are given in Tables 4 and 5. The themes and subthemes extracted are summarized in Table 6. The percentages were calculated relative to the total number of studies on each factor, by country development level.

**Table 6:** The number and percentage of most reported contributing factors of missed nursing care during the COVID‐19 pandemic by countries development status.

Country					Contributing factors to missed nursing care		
Total	Developing *N* = 31		Developed *N* = 26				
*N*	%	*N*	%	*N*			
41	60.97	25	39.02	16	Human resource allocation (insufficient number of staff)	Structure	Work environment
31	58.06	18	41.93	13	Material resources allocation (lack of medicine and PPE, lack or failure of equipment, type of ward and type of hospital)		
37	59.45	22	40.54	15	Communication, collaboration, teamwork, backup support, patient safety culture, planning	Working climate	

39	56.41	22	43.58	17	Care process (prioritization, shift handoff, nursing care model, and how to provide care), experience, expertise, competence, knowledge, skill, job burnout, type of shift, job role, employment status, degree of education, attitude and belief, motivation, responsibility, satisfaction	Individual & professional	Nurses characteristics
18	61.11	11	38.88	7	Gender, age, marriage, number of children, place of residence, number of diseases the nurse	Personal	

37	51.35	19	48.64	18	Workload, overtime work, demand for care, lack of time, fatigue, time‐consuming wearing of PPE, multitasking, number of patients under care, interruption in care, excessive documentation and duplicate, heavy admission, and discharge activity	Intensity	Workflow characteristic
28	57.14	16	42.85	12	Uncertainty in care, urgent patient situations, unpredictable situations, unexpected rise in patient volume	Predictability	
11	63.63	7	36.36	4	Stress, anxiety, fear of virus infection, spread of symptoms	Perceived risks	

Twenty five studies (60.97%) of the studies reported in developing countries considered the lack of human resources as a factor of MNC. However, only 10 studies (%24.39) [40, 44, 45, 50, 60, 68, 69, 71, 75, 76] mention this factor as the most significant MNC factor in developing countries. This is while in developed countries, 16 studies (39.02%) have pointed to the lack of human resources; however, only in 6 studies (14.63%) [47, 51, 53, 54, 67, 77], this factor is the most significant.

Examining various contributing factors in the eligible studies showed that most of the factors affecting MNC during the COVID‐19 pandemic were reported in developing countries with a higher prevalence. Therefore, it can be concluded that the level of development of countries is one of the factors involved in the occurrence of MNC during a global health crisis (Table 6).

## 5. Discussion

This hypothesis proposes that the MNC during the COVID‐19 pandemic is a multifaceted issue influenced by both internal and external factors. The findings strongly support this assumption, revealing a complex interplay across four thematic domains: workplace environment, nurse characteristics, workflow features, and the level of a country’s development. Identifying environmental factors—such as staffing shortages and workspace constraints—aligns with the structural impacts emphasized by the MNC Model, confirming that insufficient resources exacerbate the discontinuation of care. However, the significance of workflow intensity and nurse attitudes—factors intensified by the chaos of the pandemic—extends beyond traditional frameworks, indicating that the unique dynamics of crises must be integrated into existing models.

Moreover, the hypothesis is further corroborated by the differential impacts observed at various levels of national development. Studies from developing countries report higher rates of missed care attributable to structural deficiencies and perceived risks, whereas developed countries emphasize disruptions related to work intensity and nurse characteristics. This challenges simplistic assumptions that staffing shortages alone drive care reduction. A minority of studies identified human resources as the primary factor. Instead, the resilience of nurses, organizational support, and the unpredictable nature of workflow played equally vital roles, reinforcing a holistic perspective on the issue.

### 5.1. Work Environment

Work environment emerged as a central factor in our analysis, aligning with extensive prepandemic research that identified structural elements—such as staffing levels and workspace conditions—as critical determinants of nursing care quality. Kalisch et al. emphasized that staffing shortages are directly associated with missed care [1], a finding echoed in our review, where 25 studies from developing countries (60.97%) and 16 studies from developed nations (39.02%) reported human resource deficiencies. Similarly, Jones and colleagues highlighted understaffing as the primary cause of incomplete care, a trend consistently observed across the studies we analyzed [3].

However, our findings diverge by emphasizing that staffing shortages are not always the paramount factor; only 24.39% of studies from developing countries and 14.63% from developed countries identified them as the main issue. Some studies linked the successful maintenance of adequate staffing and care quality to specific factors such as the availability of adequate facilities and equipment in specialized units [54], the reduced number of routine patient admissions and lower dependency among COVID‐19 patients [84], increased managerial support for resource allocation [52], the decline in disease outbreaks across epidemic waves and hospitals’ relative preparedness, along with the postponement of elective surgeries [59], the development of personal and professional strategies to sustain care standards [74], the hospitalization of COVID‐19 patients in large hospitals equipped with sufficient resources [20], and nurses’ performance improvements informed by lessons learned from SARS and Ebola training programs [64]. These trends reflect a shift during the pandemic, where the work environment—including leadership support and organizational culture—became even more prominent as key factors influencing care delivery.

Consequently, implementing effective managerial strategies and providing adequate material resources can play a crucial role in compensating for human resource shortages and maintaining overall care quality during crises. For instance, more equipped hospital units can allocate sufficient time and resources for essential nursing activities [64]. The rate of missed care can differ based on factors such as the size of the hospital or unit [86] and the type of hospital is a significant factor in the occurrence of MNC [19]. As a result, healthcare facilities with stronger care structures and processes are better positioned to deliver superior care and achieve improved outcomes during a pandemic [87].

Furthermore, shortages of PPE and equipment contribute to heightened anxiety and fear of infection among staff [37], which leads to poor quality of care, psychological distress, burnout, and increased intentions to leave the profession [70, 88, 89] that amplify the strain on human resources during emergencies. Overall, it is evident that nurse shortages are particularly pronounced when managing high patient volumes during COVID‐19 surges and other acute crises or natural disasters. However, structural modifiable factors—such as improving physical work environments—may serve as essential elements in reducing nosocomial infection transmission (MNC) amid such crises.

A multinational study by Aiken et al. demonstrated that better work environments decrease missed care, a finding that aligns with our review, which indicates that developed countries often cite workspace quality over staffing numbers as a key concern [90]. For example, studies from Sweden and Italy identified weak organizational support as a critical motivator for missed care [32, 47], a view consistent with Aiken et al.’s emphasis on broader contextual factors beyond staffing levels. Prepandemic literature rarely addressed specific stressors of crises such as PPE shortages or increased infection control demands. Our review highlights these as amplifiers of environmental pressure, thus broadening the discussion to encompass health emergency‐related factors.

Another neglected yet influential factor during the pandemic was the work climate. Poor teamwork is a subset of the broader work environment and has been identified as a significant contributor to MNC. This observation aligns with reports from other studies [34, 35, 91–93]. Conversely, a study on team cohesion during COVID‐19 reported positive outcomes [94], emphasizing that the main challenges to fully realizing nurses’ roles stem from lack of support from team members, trust deficits, and communication disruptions within nursing teams [14, 48, 95]. Consequently, teamwork and nurses’ clinical leadership skills directly influence missed care [96]. According to Kalisch’s model, interpersonal relationships and teamwork are critical external factors affecting nursing care [1]. Kim et al. [97] described MNC as a specific outcome impacted by nurses’ work environment and patient safety culture [97].

Therefore, an improved work environment for nursing forms the foundation for delivering high‐quality care, which can ultimately enhance job satisfaction, foster a positive safety climate, and reduce neglect of necessary care routines [98, 99]. Additionally, nurses’ positive attitudes toward patient safety are associated with reductions in missed care activities [100]. Communication is an essential part of patient care in times of crisis. Combining listening skills and emotional intelligence with training in communication management and conflict resolution skills has been suggested to improve perceived team communication [101]. Implementing management solutions, such as the promotion of professional empowerment, the enhancement of nurses’ job satisfaction, and the balancing of nurses’ rights, can effectively reduce or prevent the occurrence of MNC [102]. It is imperative for nurse managers to assess both the work environment and MNC as key factors in predicting and improving patient care quality.

### 5.2. Nurse Characteristics

Our findings highlight that nurse characteristics—including professional competencies, and personal factors, such as burnout, attitude and belief, motivation, responsibility, and satisfaction—are significant determinants, aligning with previous research [1, 103], while also revealing subtle, pandemic‐specific nuances. Kalish et al. also mentioned the elements related to the internal processes of nurses (group and team norms, prioritizing decisions, internal values ​​and beliefs, and habits). If the existence of unfavorable reasons overcomes the internal processes of nurses, MNC occurs [1]. For instance, Srulovici et al. demonstrated that nurses’ perceptions of workload and professional values influence their care delivery, a pattern reflected in our review where attitudes toward prioritization significantly shape the extent of missed care [104]. However, personal factors like fear of infection and emotional exhaustion emerged prominently during the pandemic, consistent with the large‐scale study by Liu et al. on frontline nurses in Wuhan, which reported increased burnout and anxiety levels [105]. A qualitative study of nurses’ experiences during the COVID‐19 period revealed that nurse assumptions and nurse bias contributed to MNC [106]. If the attitude of nurses toward patient safety is positive, MNC will decrease [100]. An observational study by Lasater et al. conducted during COVID‐19 further supports these insights by linking chronic staffing shortages and pandemic‐induced pressures with heightened burnout, which in turn increases missed care [107]. Prepandemic literature rarely emphasized such psychological stressors, as prior focus primarily centered on stable healthcare environments. Therefore, incorporating fear and resilience as key nurse‐related factors adds a critical crisis dimension, underscoring the necessity of emotional and mental health support alongside traditional competency training. This approach is grounded in Liu et al.’s framework, which incorporates personal resilience as a moderator in delivering care during emergencies [108]. In a study conducted in Australia, Cyprus, and Italy, factors such as nurses’ age (younger), highest degree attained, absenteeism, workplace type, clinical experience, and clinical competence explained variance in missed care scores [107]. The younger age of nurses is associated with lower qualifications and less clinical experience. Concurrently, managing complex crises requires proficiency in performing multiple tasks simultaneously and effectively utilizing personal and professional strategies; otherwise, a higher proportion of care is likely to be missed. Other studies have also identified the use of personal and professional strategies as protective factors against MNC [104, 109].

### 5.3. Workflow Characteristics

Workflow characteristics—namely, intensity, predictability, and risk—represent a relatively new area within our review, although they resonate strongly with the broader healthcare literature concerning disruptions. Abbaszadeh et al. work on workflow interruptions linked these disturbances to care errors, a concept that our analysis extends to include missed care during the pandemic [110]. Qualitative studies within our sample [32–35] highlighted the unpredictability and high‐risk nature of COVID‐19 care—factors like rapid patient deterioration and isolation protocols—as key drivers of missed tasks. This contrasts with prepandemic literature, where workflow was seldom a primary focus in models explaining missed care, such as the traditional MNC framework.

Zabaleta et al.’s scoping review of MNC during COVID‐19 identified increased workload but did not explicitly categorize workflow dynamics as we did, particularly regarding intensity, predictability, and risk [17]. Similarly, Labrague et al.’s systematic review noted increased task demands but lacked detailed thematic differentiation on the severity and risk factors outlined here [16]. Additionally, Heng et al. referred to the unpredictability of the work environment and increased workload as significant contributors to MNC [89]. A study in Switzerland showed that during the COVID‐19 outbreak, organizational changes, feelings of fear, and uncertainty significantly impacted nurses and patient care [13]. In this context, it is also important to consider the care of those who provide care, which should be planned and implemented in different scenarios [111]. Providing informed management support in crises and improving nurses’ working environments along with disseminating evidence‐based information to nurses, in addition to reducing stress and anxiety related to care in emergencies, ensures sufficient time to provide care.

### 5.4. Country Development Level

The influence of a country’s level of development on MNC constitutes a unique aspect of this review, as it directly links broader health system inequalities with the increased occurrence of missed cares. While Kruk et al.’s analysis of global health systems has documented resource disparities particularly affecting low‐ and middle‐income countries [112], previous studies have shown that MNC is more prevalent in low‐income countries compared to high‐income ones [19]. Notably, middle‐ and low‐income countries face specific structural deficiencies within their healthcare systems [18]. Conversely, high‐income nations allocate substantial portions of their budgets to support healthcare systems [113]. Thus, a more favorable work environment correlates with higher nurse satisfaction and correspondingly less missed care [114]. Consequently, systemic vulnerabilities may amplify missed care during crises, emphasizing the importance of resilient healthcare infrastructure and supportive work climates to mitigate such risks.

Our study explicitly associates these disparities with higher rates the nature of factors that lead to MNC. Developing nations primarily reported structural deficiencies such as staffing shortages and equipment limitations, with 60.97% of studies referencing human resources. Conversely, high‐income countries like Sweden and Poland emphasized workflow issues and nurse‐related concerns; only 39.02% of their studies pointed to staffing deficits. Fewer (23.39% of studies from developing countries and 14.63% of studies from developed countries) ranked these as the most significant factors.

This finding diverges from the prepandemic literature, which rarely framed missed care through a development perspective and instead focused on global factors such as nurse‐to‐population ratios [1, 3]. In a systematic review from 2017 to 2021, five factors associated with the phenomenon of MNC were reported: lack of job satisfaction, nurse staffing shortage, nurse competence level, the care environment, and nurses’ workload/burnout. These findings partly support our study results [115]. The systematic review by Bayram et al., analyzing 25 studies during COVID‐19, identified regional variations but prioritized emotional and psychological well‐being over systemic inequalities [15]. Consequently, our study advances understanding by quantitatively demonstrating how macrolevel socioeconomic contexts shape care delivery, filling a crucial gap and aligning with Kruk et al.’s call for health system reforms tailored to specific contexts [112]. This perspective challenges the universal applicability of Western‐centric models and emphasizes the importance of context‐appropriate interventions in resource‐limited settings.

A comparison of our findings with other COVID‐19‐specific reviews reveals both points of convergence and divergence. For example, Zabaleta et al.’s review, which examined 25 studies up to March 2023, quantified the phenomenon of incomplete care but provided less thematic depth than our four‐category framework [17]. The smaller review by Labrague et al. (10 studies) documented reasons for missed care consistent with themes related to work environment and nurse characteristics but overlooked the influence of systemic development levels and workflow issues during crises [16]. The systematic review by Chiappinotto et al., which analyzed five studies across different pandemic waves, supported our findings regarding staff support but did not explore broader systemic factors or crisis‐specific workflow challenges [21]. Similarly, the integrated review conducted by Sist et al., encompassing four studies, identified systemic and nurse‐related factors consistent with our themes but lacked insights into workflow challenges particular to emergency situations [22]. Furthermore, focusing on emotional and psychological aspects, Bayram et al. reinforced the importance of nurse well‐being but underestimated the broader structural and workflow elements critical to understanding missed care in crisis contexts [15].

This comprehensive review both confirms and extends existing literature, emphasizing the central roles of the work environment and nurse characteristics, while also highlighting workflow issues and developmental disparities as key factors during crises. While the prepandemic literature primarily identified core factors such as staffing levels and the work environment [1, 3, 90, 116], it rarely addressed global crises or developmental inequalities. Studies focusing specifically on the pandemic era [15–17, 21, 22] advanced this field by documenting instances of missed care during COVID‐19; however, their scope or focus was limited, creating gaps in thematic synthesis and cross‐national comparison. Our study fills these gaps by offering a robust, multidimensional analysis that integrates microlevel perspectives (nurse experiences, workflow) with macrolevel considerations (national development), thus providing a more comprehensive understanding of MNC.

The strength of our review lies in its broad scope—examining 57 studies—and its incorporation of pandemic‐specific factors, which extend beyond the narrower samples or limited focus of prior research. It confirms that staff stress and burnout are global issues while highlighting workflow disruptions and developmental inequalities as critical moderators during a crisis, thereby enriching the ongoing discourse.

Our findings align with the conceptual framework of MNC, which posits those structural factors (work environment) and process elements (workflow, nurse characteristics) drive missed cares [1]. However, the pandemic context necessitates adaptation: the intensity of workflow demands and the level of national development emerge as dynamic variables absent from conventional formulations. This reflects process‐oriented insights similar to those of Tucker and Edmondson, as well as the macrolevel perspectives of Kruk et al., leading us to propose a hybrid model that considers crisis‐specific stressors [112, 117]. Foundational studies by Kalisch et al. and Jones et al. provide a platform, but our review expands their applicability to emergency scenarios [1, 3]. These findings necessitate interventions aimed at addressing staffing shortages, supply chain resilience, communication systems, and nurses’ well‐being—particularly in resource‐constrained settings—to reduce the extent of missed cares during future health crises.

## 6. Limitation

This scoping review aims to map the landscape of factors influencing MNC during the pandemic across a broad geographic scope, drawing on data from 23 countries. Several limitations should be kept in mind when interpreting the findings:

Scope and purpose of a scoping review: Our objective is to chart existing research, identify gaps, and outline directions for future work rather than quantify effect sizes or establish causal relationships. As a result, the review is not designed to make strong inferences about the magnitude or direction of associations between factors.

Analytical limitations: While our thematic analysis provides detailed insights, it remains subject to the inherent interpretive nature of qualitative synthesis.

Search and indexing limitations: Despite using comprehensive search terms, the absence of controlled vocabulary indexing may have caused us to miss some relevant studies.

Implications for interpretation: Given the breadth and diversity of the evidence base, findings should be viewed as a landscape rather than a definitive guide. Cautious interpretation is warranted, and the results should be used to inform targeted hypotheses and future, more rigorous research designs.

Addressing these limitations—through longitudinal designs, standardized definitions and measures, preregistered protocols, and systematic indexing—would strengthen the reliability and applicability of future research on MNC in crisis contexts.

## 7. Conclusion

This review systematically synthesizes data from 57 studies conducted worldwide to identify key factors influencing MNC during the COVID‐19 pandemic. It advances previous reviews by introducing a novel four‐domain conceptual framework encompassing the work environment, nurse‐related factors, workflow characteristics, and the country’s level of development. By highlighting workflow as an independent determinant and comparing contexts across development levels, this review provides a more integrative understanding of the systemic and contextual drivers of MNC. Variations in healthcare systems and hospital structures between developed and developing countries significantly affect the specific challenges and operational needs encountered within each context. The findings underscore an urgent necessity for the implementation of contextually appropriate interventions aimed at mitigating MNC, enhancing patient care quality, and strengthening the resilience of healthcare systems in preparation for future crises.

Furthermore, this study highlights the crucial role of a robust and well‐developed conceptual framework that can serve as an effective tool for understanding and managing MNC during periods of crisis. Adopting this comprehensive approach not only facilitates a more profound understanding of existing issues but also supports the development of more targeted and effective strategies for future emergency preparedness.

Given the increased involvement of nursing staff during pandemics, missed care becomes particularly critical at the microlevel—specifically, the nurse–patient interface. Caring for an entirely new patient population requires the acquisition of new skills to adapt to the challenges. A promising avenue for future research involves developing AI‐based predictive models that analyze multidimensional, real‐time data—such as workload, environmental conditions, and behavioral indicators—to estimate the psychological and professional attributes of nurses. While these models can approximate stress levels and behavioral tendencies, capturing deep‐seated beliefs, attitudes, and professional characteristics as well as perspectives of patients requires integrating qualitative assessments. Such proactive tools could identify hidden contributors to missed cares, supporting more targeted and effective crisis management. Without strategic planning and adequate preparedness, healthcare systems remain vulnerable to repeated occurrences of missed care, which threaten patient safety and operational efficiency.

## 8. Implications for Nursing Managers and Policymakers

The findings of this review demonstrate that MNC during health crises arises from the interaction of organizational structures, nurse characteristics, workflow intensity, and health system capacity. To address this, nursing managers should prioritize strategic workforce planning, ensuring flexible staffing models and surge capacity preparedness. Strengthening the work environment through resource availability, effective communication, and a supportive safety culture can help mitigate crisis pressures.

Policymakers should focus on investing in workforce retention, crisis‐specific training, and workflow redesign to enhance adaptability and reduce risk. Importantly, addressing structural disparities between health systems and embedding nurse leaders in decision‐making processes will ensure that operational realities inform national preparedness strategies. Tailored approaches are particularly essential, as developing countries require fundamental infrastructure and workforce expansion, whereas developed countries may benefit more from organizational adaptability and workforce well‐being initiatives.

By aligning these recommendations with the identified themes, healthcare systems can enhance the quality of nursing services. These actions can optimize the delivery of functional and patient‐centered care while improving global outcomes (e.g., reducing disease spread), organizational outcomes (e.g., improving quality and safety), patient outcomes (e.g., better prognosis), and nursing outcomes (e.g., greater satisfaction, reduced burnout, and lower turnover intentions) during critical situations.

## Conflicts of Interest

The authors declare no conflicts of interest.

## Funding

The Student Research Committee of Tabriz University of Medical Sciences financially supported this research.

## Data Availability

The data supporting the findings of this study are available from the corresponding author upon reasonable request.
