# Correction to “Mapping the Contributing Factors to Missed Nursing Care in Hospital Settings During a Global Health Crisis: A Systematic Scoping Review”

**DOI:** 10.1155/jonm/9760503

**Published:** 2026-05-17

M. Pourshaban, A. Allahbakhshian, and M. Purabdollah, “Mapping the Contributing Factors to Missed Nursing Care in Hospital Settings During a Global Health Crisis: A Systematic Scoping Review,” *Journal of Nursing Management*, 2025, 7343469, https://doi.org/10.1155/jonm/7343469.

In the article, the incorrect affiliation was given for Mahsa Pourshaban. This has been corrected from:

• Department of Medical‐Surgical Nursing, Faculty of Nursing and Midwifery, Tabriz University of Medical Sciences, Tabriz, East Azerbaijan Province, Iran

to:

• Student Research Committee, Tabriz University of Medical Sciences, Tabriz, Iran

Additionally, information was erroneously omitted from the funding statement, and has now been updated to the below:

• “The Student Research Committee of Tabriz University of Medical Sciences financially supported this research”

Lastly, the acknowledgements section has been updated from:

• “The authors would like to thank the Department at the Tabriz University of Medical Sciences, who provided great help in accessing the databases.”

to:

• “The authors would like to thank the Student Research Committee of Tabriz University of Medical Sciences for their valuable support. Grant Number: 75775.”

We apologize for these errors.

